# Visualizing the Integrity of Chloroplast Envelope by Rhodamine and Nile Red Staining

**DOI:** 10.3389/fpls.2021.668414

**Published:** 2021-04-26

**Authors:** Jinjie An, Xin Miao, Lulu Wang, Xu Li, Xiaomin Liu, Hongbo Gao

**Affiliations:** ^1^Beijing Advanced Innovation Center for Tree Breeding by Molecular Design, Beijing Forestry University, Beijing, China; ^2^College of Biological Sciences and Technology, Beijing Forestry University, Beijing, China

**Keywords:** chloroplast isolation, chloroplast integrity, envelope, staining, Rhodamine, Nile red

## Abstract

Chloroplasts are essential organelles in plant cells with many important functions. Chloroplasts isolated by Percoll density gradient centrifugation are widely used in the study of chloroplasts. The intactness of isolated chloroplasts is necessary for many of the experiments. In the past, those isolated chloroplasts were either simply believed to be intact or had to be analyzed by indirect biochemical methods. Here we show a new method to check the intactness of isolated chloroplasts by staining their envelope with fluorescent dyes, Rhodamine or Nile red, and then observing them with a fluorescence microscope. With this method, broken chloroplasts and intact chloroplasts can be distinguished easily and their integrity can be checked in a few minutes. Results of this method agreed well with those of biochemical methods. Moreover, we have also found that sometimes the middle layer chloroplasts from the Percoll gradient centrifugation could be mostly broken, which could cause mistakes in the experiment. With our method, this problem can be easily found. This chloroplast envelope staining method can be used in the preparation of isolated chloroplasts to ensure the intactness.

## Introduction

Chloroplasts are plant-specific organelles encapsulated by double membranes, inside which contain thylakoids, stroma, and other structures. Chloroplasts are the major site of photosynthesis. They are also involved in many other important biochemical processes in plants, such as the biosynthesis of fatty acids, amino acids, and plant hormones (Azevedo et al., 2006; Lopez-Juez, 2007; Schaller and Stintzi, 2009; Sendon et al., 2011; Mano and Nemoto, 2012; Wang and Benning, 2012). Chlorophyll in the thylakoids of chloroplasts can emit autofluorescence upon excitation (Ruhle et al., 2018), which is often used for the observation chloroplasts with fluorescence microscope (Gao et al., 2013).

Biochemical, physiological, and proteomic analyses are common methods to study the functions and development of chloroplasts (Gao et al., 2003, 2006; Baginsky and Gruissem, 2004; Block et al., 2007; Lopez-Juez, 2007; Su and Lai, 2017; Fuertauer et al., 2019). During the process of these analyses, chloroplasts are often isolated and the intactness of chloroplasts are required to maintain their metabolic activity and the functional surrounding envelope membrane at the maximum (Seigneurin-Berny et al., 2008b; Su and Lai, 2017). The density of chloroplasts will be decreased once they are broken. Due to the difference of the densities between broken chloroplasts and intact chloroplasts, Percoll density gradient centrifugation method is widely used to separate broken chloroplasts and obtain pure and intact chloroplasts (Fitzpatrick and Keegstra, 2001; Seigneurin-Berny et al., 2008a). After centrifugation, broken chloroplasts usually stay in the upper layer, and intact chloroplasts usually stay in the middle layer between the Percoll of two different densities.

In the past, it was generally believed that chloroplasts obtained by Percoll density gradient centrifugation are intact (Seigneurin-Berny et al., 2008b; Lang et al., 2011; Vieira Ldo et al., 2014), but this may not always be true. During the day, starch granules are accumulated in chloroplasts, which not only make chloroplasts fragile during the isolation process but also increase the density of chloroplasts. Therefore, broken chloroplasts with high starch content may stay in the middle layer of Percoll gradient and misled the experiment. The integrity of chloroplasts can be analyzed by SDS-PAGE analysis (Seigneurin-Berny et al., 2008a,b). However, it will take hours to 1 day get the final results. Hill reaction is a quick method widely used to check the intactness of chloroplasts (Mills and Joy, 1980). It estimates the intactness of chloroplasts based on biochemical reactions indirectly. Direct observation with bight field, phase contrast, and autofluorescence are difficult to discern broken chloroplasts with good shapes. In this study, we developed a new method using fluorescent dyes, such as Rhodamine or Nile red, to stain the envelop membrane of isolated chloroplasts and then directly check their integrity by microcopy observation. This method is very simple. With this method, we found that isolated chloroplasts from the middle layer of Percoll gradient are not totally intact and this can be quickly observed with a fluorescence microscope.

## Materials and Methods

### Plant Materials and Growth Conditions

*Arabidopsis thaliana* plants used are Columbia-0 (Col) wild type. Plants were grown in soil in a growth chamber at 22°C with 40–60% relative humidity and 16-h-light/8-h-dark cycles. Tobacco (*Nicotiana benthamiana*) and poplar (*Populus alba* × *Populus tremula var. glandulosa*) plants were grown under the same growth condition. Tobacco plants were 5-week-old and poplar plants were 6-month-old. Spinach, pea and lettuce were bought from a vegetable shop. *Peperomia tetraphylla* and orchid were grown on a sunny windowsill at room temperature and 6-month-old.

### Chloroplast Isolation

All the steps were carried out at 4°C. For each sample, 7 g leaves were harvested, put into a blender, and added with 100 mL GB solution [50 mM HEPES/KOH (pH 7.9), 0.33 M sorbitol, 1 mM MgCl_2_, 1 mM MnCl_2_, 2 mM EDTA (pH8.0), 5 mM Na-ascorbate, 0.1% BSA]. These leaf tissues was ground five times, 3 s each time. The ground samples were filtered into 50 mL tubes, centrifuged at 2000 *g* for 2 min. After removing the supernatant, 500 μL RB solution [50 mM HEPES-KOH (pH 7.9), 0.33 M sorbitol, 0.8 mM CaCl_2_, 5 mM Na-ascorbate] was added to the chloroplast pellet. The chloroplasts were resuspended and carefully added to the surface of a Percoll density gradient solution in RB with 40% (v/v) Percoll on the top, and 90% (v/v) Percoll at the bottom. After a centrifugation at 2000 *g* for 10 min, chloroplasts were separated by Percoll gradient into upper layer and middle layer. For washing, chloroplasts from each layer were carefully transferred into a new tube and resuspended with 3 mL RB, centrifuged at 400 *g* for 3 min, and resuspended in RB again.

### Chloroplast Staining

1 μL Rhodamine B (0.1 mg/mL) or Nile red (1 mg/mL) were added into 100 μL chloroplast suspension. Then the chloroplasts were stained on ice for 2–10 min. After being collected by centrifugation at 3000 *g* for 7 s and washed with RB twice, chloroplasts were observed with a fluorescence microscope.

### Microscopy and Image Analysis

Isolated chloroplasts were observed with a NEXCOPE NE910 microscope equipped with 40 × and 20 × objectives, and images were captured with an E3ISPM camera. Autofluorescence of chlorophyll, and red fluorescence of Rhodamine and Nile red were observed with an excitation filter set of 510–580 nm and an emission filter set of 600–700 nm. Image analysis was carried out using ImageJ (http://rsbweb.nih.gov/ij/; version 1.52v) and Photoshop (Adobe Photoshop CC 2015).

### Statistical Analysis of Chloroplast Integrity

For each stained chloroplast sample of the middle layer, 20 fields observed with 40 × objective were taken to count the intact and broken chloroplasts. The ratio of intact or broken chloroplasts to the total chloroplasts (*n* > 400) was calculated. Data was analyzed by Excel.

### Coomassie Blue Staining and Immunoblotting

Proteins were extracted from the leaves, chloroplasts of the upper layer and the middle layer, respectively. Protein samples with equal amount of chlorophyll were loaded in each lane, resolved by SDS-PAGE, stained in Coomassie brilliant blue solution (1 g Coomassie brilliant blue R250, 200 mL ethanol, 100 mL acetic acid, add deionized water to 1 L) for 4 h. Subsequently, the gel was destained in destaining solution (200 mL ethanol, 100 mL acetic acid, add deionized water to 1 L) for several hours. Protein samples resolved by SDS–PAGE gels were also transferred to Immun-Blot PVDF membrane (Bio-Rad). Blots were probed with anti-TOC75 primary antibodies (Chen et al., 2016) (1:40,000 dilution) for 1 h and HRP conjugated anti-rabbit secondary antibodies (1:10,000 dilution) for 1 h. Films were developed using eECL Western Blot Kit (Beijing ComWin Biotech Company).

## Results

### Conventional Microscopy Method Is Not Good at Discerning Broken and Intact Chloroplasts

Chloroplast isolation is a common experiment in many labs. Obtaining intact chloroplasts is important for the study of chloroplasts. Isolated chloroplasts can be observed with a bright field, phase contrast or fluorescence microscope to check their integrity. If a chloroplast is highly disintegrated, its shape will be abnormal and can be easily recognized. However, it is difficult to directly observe the integrity of isolated chloroplasts with broken envelope and relatively good shapes (Supplementary Figure 1). In some cases (see below), a majority of isolated chloroplasts could be broken with unawareness and this could cause a serious problem to the experiment.

### Rhodamine Can Be Used to Discern Broken and Intact Chloroplasts by Staining Their Envelope

**Figure 1:**
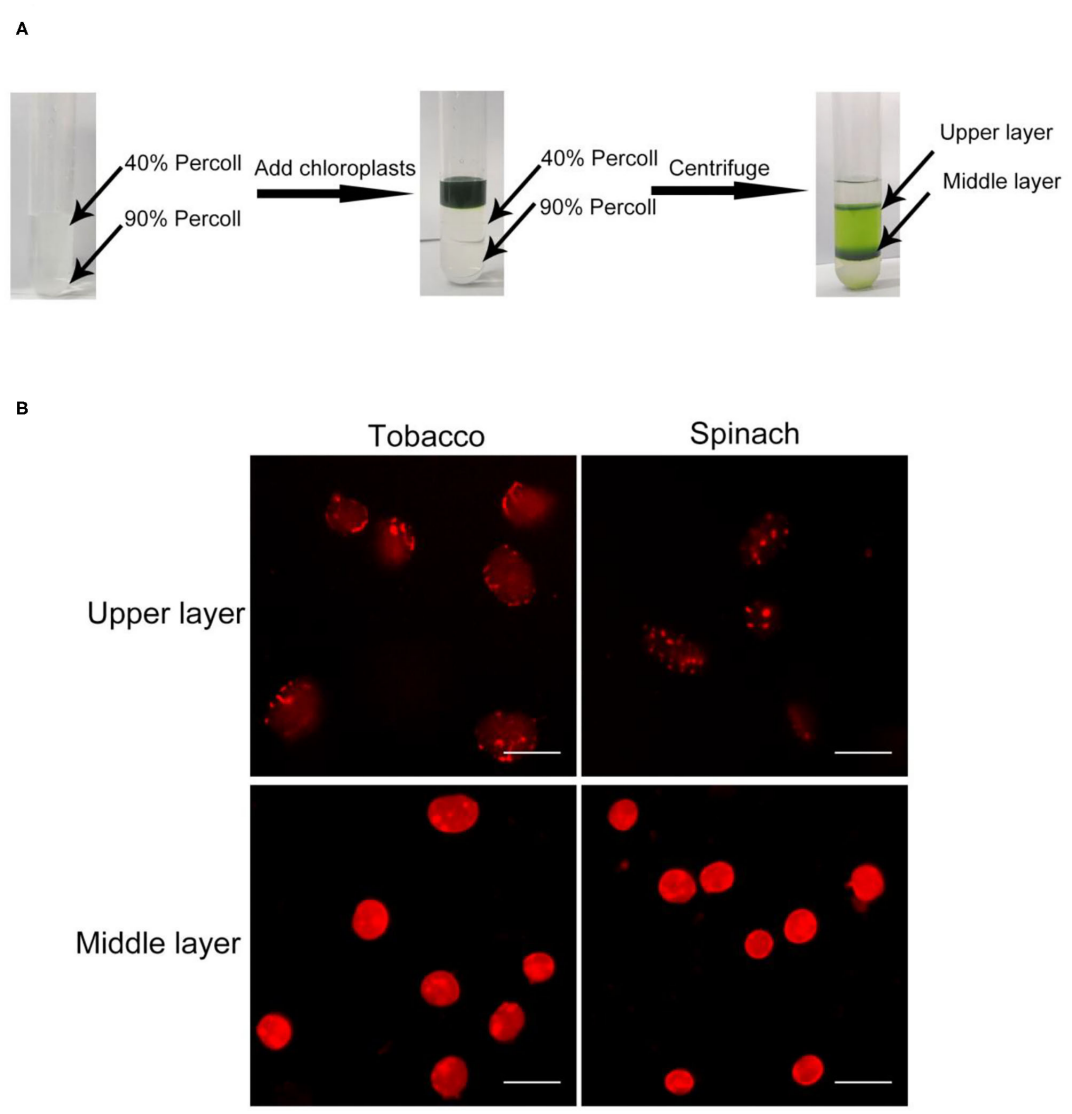
Chloroplast isolation and Rhodamine staining. **(A)** A diagram of the isolation of chloroplasts by Percoll density gradient centrifugation. Crude chloroplasts were collected and loaded onto the top of a Percoll gradient. After centrifugation, chloroplasts usually will be separated into the upper layer and the middle layer. **(B)** Rhodamine staining of chloroplasts. Chloroplasts from tobacco and spinach were separated by steps shown in **(A)**, stained with Rhodamine and observed with a fluorescence microscope. Bar = 10 μm.

In order to discern intact chloroplasts and broken chloroplasts, we developed a method to evaluate the intactness of isolated chloroplasts. At first, crude chloroplasts isolated from plant leaves were applied to a Percoll density gradient centrifugation to get the upper layer chloroplasts and middle layer chloroplasts (Figure 1A). These chloroplasts were stained with Rhodamine for 2–10 min and washed twice. Rhodamine is a dye with red fluorescence under the excitation of a 550 nm laser (Chen and Wood, 2009), and it can bind to chloroplast envelope and show the integrity through microscopy observation (Figure 1B).

In the beginning, we isolated chloroplasts from tobacco and spinach leaves, and checked the integrity of isolated chloroplasts with the above method. After staining, the envelope of chloroplasts can be visualized clearly (Figure 1B). For most of the chloroplasts from the upper layer, the envelope was discontinuous and apparently broken. In contrast, for most of the chloroplasts from the middle layer, the envelope was continuous and obviously intact. This staining method allowed us to visually identify intact and broken chloroplasts.

### Rhodamine Staining Is a Reliable Method to Check the Integrity of Chloroplasts

**Figure 2:**
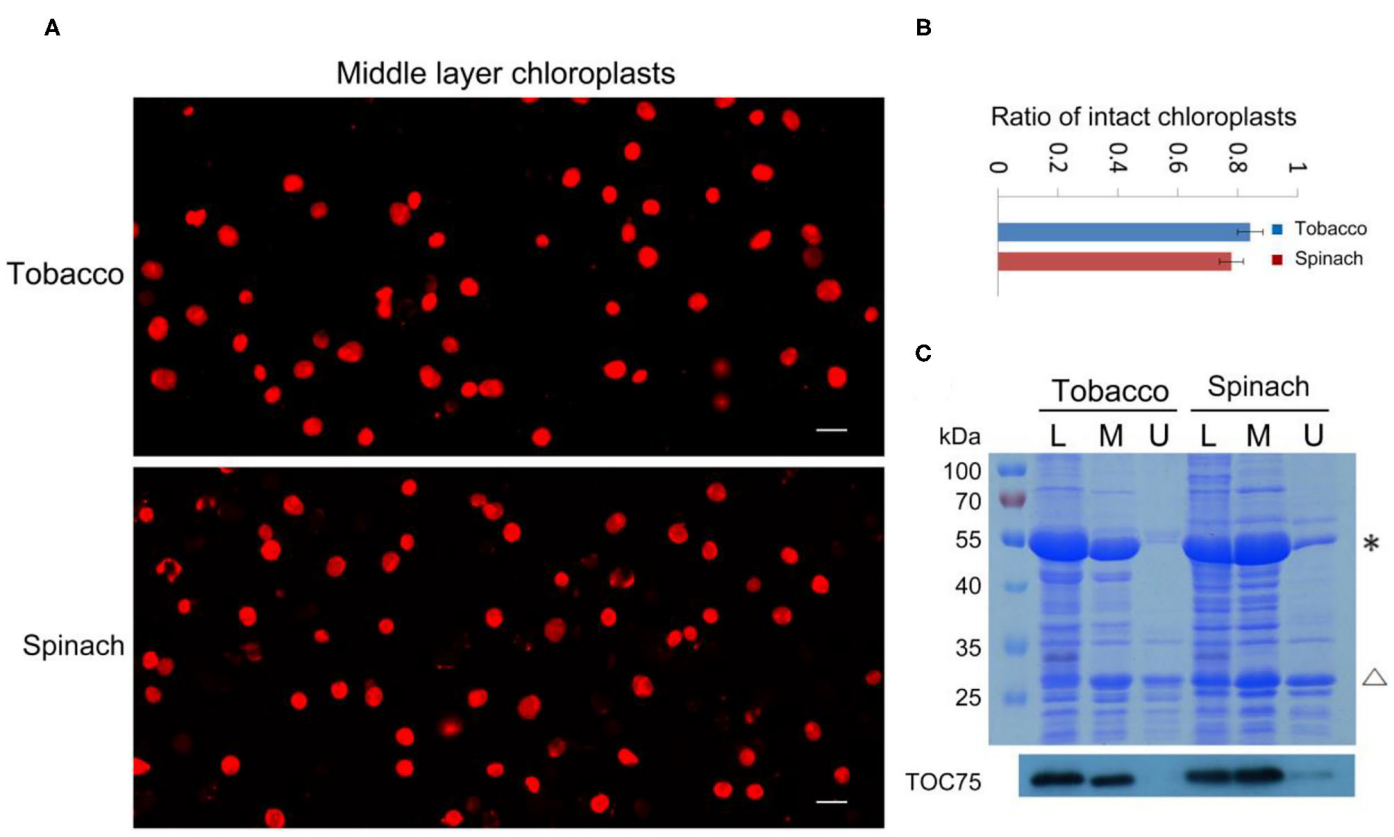
Analysis of the integrity of isolated chloroplasts from tobacco and spinach. **(A)** Chloroplasts from the middle layer of tobacco and spinach were stained with Rhodamine and then observed with a fluorescence microscope. Bar = 10 μm. **(B)** Statistical analysis of the proportion of intact chloroplasts. Chloroplasts from the middle layer of tobacco and spinach were photographed and analyzed. Error bars represent standard deviation. **(C)** The intactness of chloroplasts from tobacco and spinach was analyzed by SDS-PAGE and Immuno-blot. Total proteins were extracted from leaves (L), chloroplasts of the middle layer (M), and chloroplasts of the upper layer (U) in tobacco and spinach. Proteins with equal amount of chlorophyll were loaded in each lane. These samples were resolved by SDS-PAGE, stained with Coomassie blue (the upper image), and detected by Toc75 antibodies through immuno-blot (the lower image). *, Rubisco; Δ, LHCII. The molecular weight of protein markers is labeled on the left.

With our method, the ratio of intact and broken chloroplasts could be calculated in a relatively precise way (Figures 2A,B). It indicated that 84 and 78% of the middle layer chloroplasts from tobacco and spinach were intact, respectively. To further verify the effectiveness of this method, we carried out SDS-PAGE, Coomassie Blue staining and immuno-blot analysis. Proteins of the leaves, upper layer chloroplasts and middle layer chloroplasts from tobacco and spinach were extracted for comparison (Figure 2C). Rubisco is a key enzyme for carbon fixation in photosynthesis, it is a soluble and highly abundant protein localized to the stroma of chloroplasts (Suzuki et al., 2010). LHCII is a key protein for light harvesting in photosynthesis, it is also highly abundant and localized to the grana of thylakoids (Gao et al., 2018; Liu et al., 2019). If the envelope of a chloroplast is broken, its Rubisco will be mostly lost, but its LHCII can be mostly kept. So, the integrity of isolated chloroplasts can be deduced by comparing the ratio of Rubisco and LHCII to that of the leaves. As we can see from Figure 2C, the Rubisco from the middle layer chloroplasts of tobacco and spinach were close to those of leaves, while the Rubisco from the upper layer chloroplasts were very low compared with those of leaves, suggesting that the chloroplasts from the middle layer were mostly intact and the chloroplasts from the upper layer were mostly broken. Toc75 is an outer envelope membrane protein of chloroplasts (Chen et al., 2016), it can also be used to reveal the integrity of isolated chloroplasts. Our immuno-blot assay results indicated that Toc75 level in the middle layer chloroplasts was close to that of the leave, while its level in the chloroplasts of upper layer was very low (Figure 2C), suggesting that the envelope of the middle layer chloroplasts was mostly kept and intact, and the envelope of the upper layer chloroplasts was mostly broken and lost. These results are consistent with each other, suggesting that Rhodamine staining method is good for evaluating the intactness of isolated chloroplasts.

### Rhodamine Can Be Used to Check the Integrity of Chloroplasts Isolated From Various Plants

**Figure 3:**
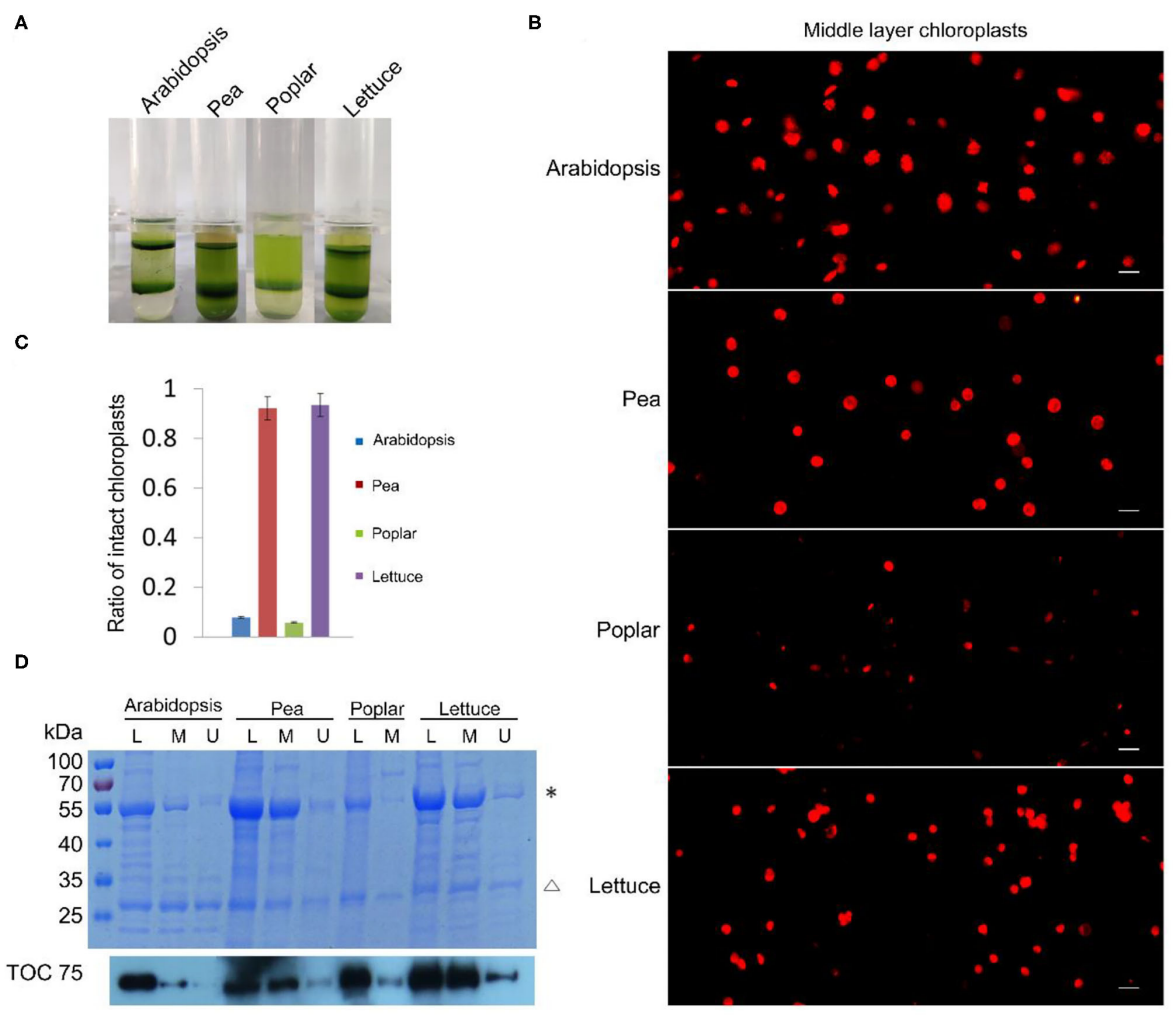
Analysis of the intactness of isolated chloroplasts from Arabidopsis, pea, poplar, and lettuce. **(A)** Chloroplasts were separated into upper layer and middle layer by Percoll density gradient centrifugation. **(B)** Chloroplasts from the middle layer in **(A)** were stained with Rhodamine and then observed with a fluorescence microscope. Bar = 10 μm. **(C)** Statistical analysis of the proportion of intact chloroplasts. Chloroplasts from the middle layer were photographed and analyzed. Error bars represent standard deviation. **(D)** The integrity of chloroplasts was analyzed by SDS-PAGE and Immuno-blot. Total proteins were extracted from leaves (L), chloroplasts of the middle layer (M), and chloroplasts of the upper layer (U). Proteins with equal amount of chlorophyll were loaded in each lane. These samples were resolved by SDS-PAGE, stained with Coomassie blue (the upper image), and detected by Toc75 antibodies through immuno-blot (the lower image). *, Rubisco; Δ, LHCII. The molecular weight of protein markers is labeled on the left.

Besides tobacco and spinach, we also tested our method with the chloroplasts isolated from other plants. We isolated chloroplasts from Arabidopsis, pea, poplar, and lettuce, stained the chloroplasts from the middle layer, and observed them with a fluorescence microscope (Figure 3A). A majority of the chloroplasts from pea and lettuce were intact. But surprisingly, chloroplasts from the middle layer of Arabidopsis and poplar looked to be mostly broken. We further calculated the proportion of intact chloroplasts to total chloroplasts of these plants (Figure 3B). The results indicated that ratios of intact chloroplasts from pea and lettuce were 92 and 93%, whereas those of Arabidopsis and poplar were only 8 and 6%, respectively (Figure 3C). Although a large part of the chloroplasts from Arabidopsis were well-stained, their envelopes were not smooth, suggesting they were fragile and mostly broken. Moreover, we used Coomassie blue staining and immuno-blot methods to analyze the intactness of chloroplasts from the middle layer (Figure 3D). The conclusions were similar to that of Rhodamine staining, further suggesting that our method is reliable and it can be applied to other plants.

### Chloroplast Envelope Integrity Can Also Be Visualized by Nile Red Staining

**Figure 4:**
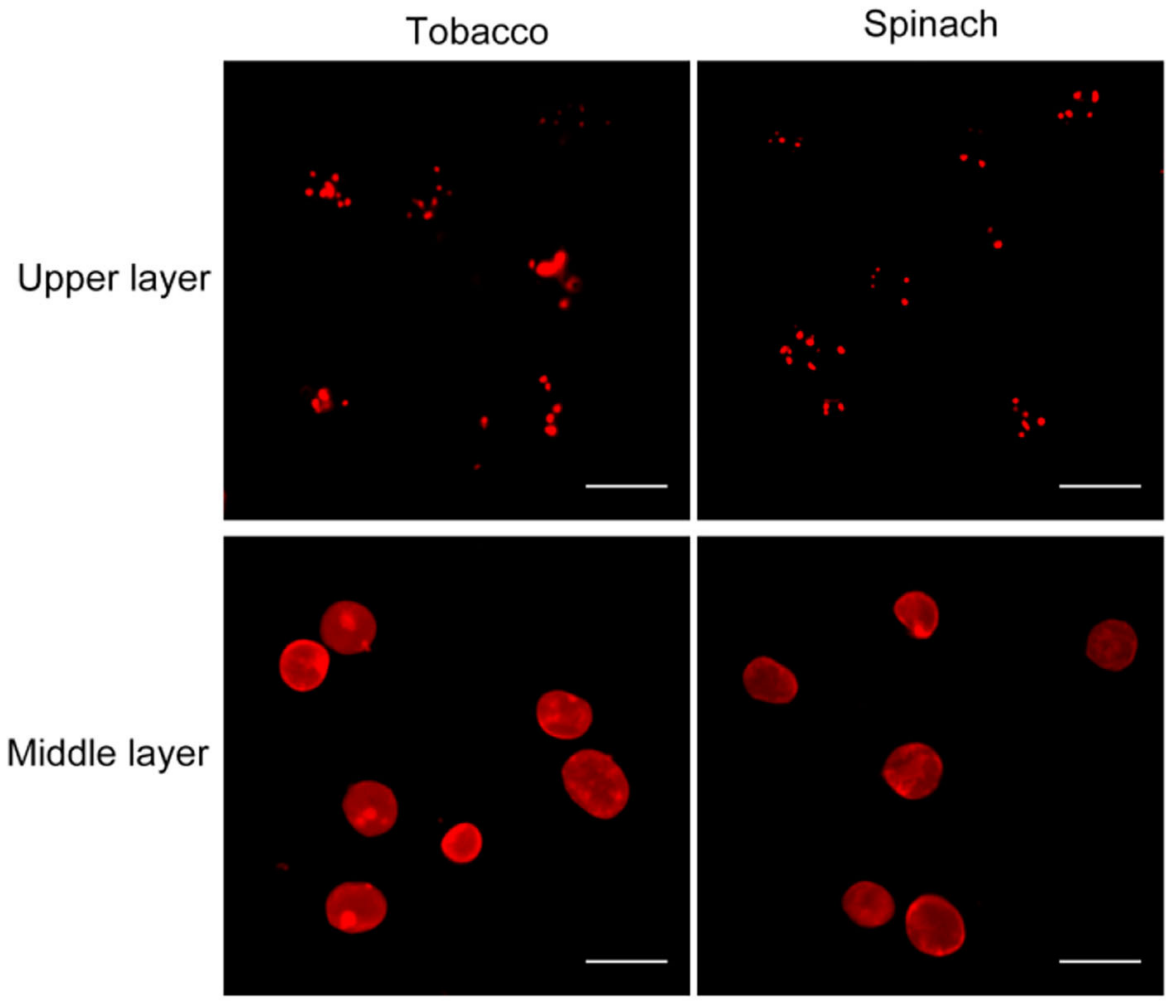
Nile red staining of chloroplasts. Chloroplasts from tobacco and spinach were isolated by steps shown in Figure 1A, stained with Nile red and observed with a fluorescence microscope. Bar = 10 μm.

**Figure 5:**
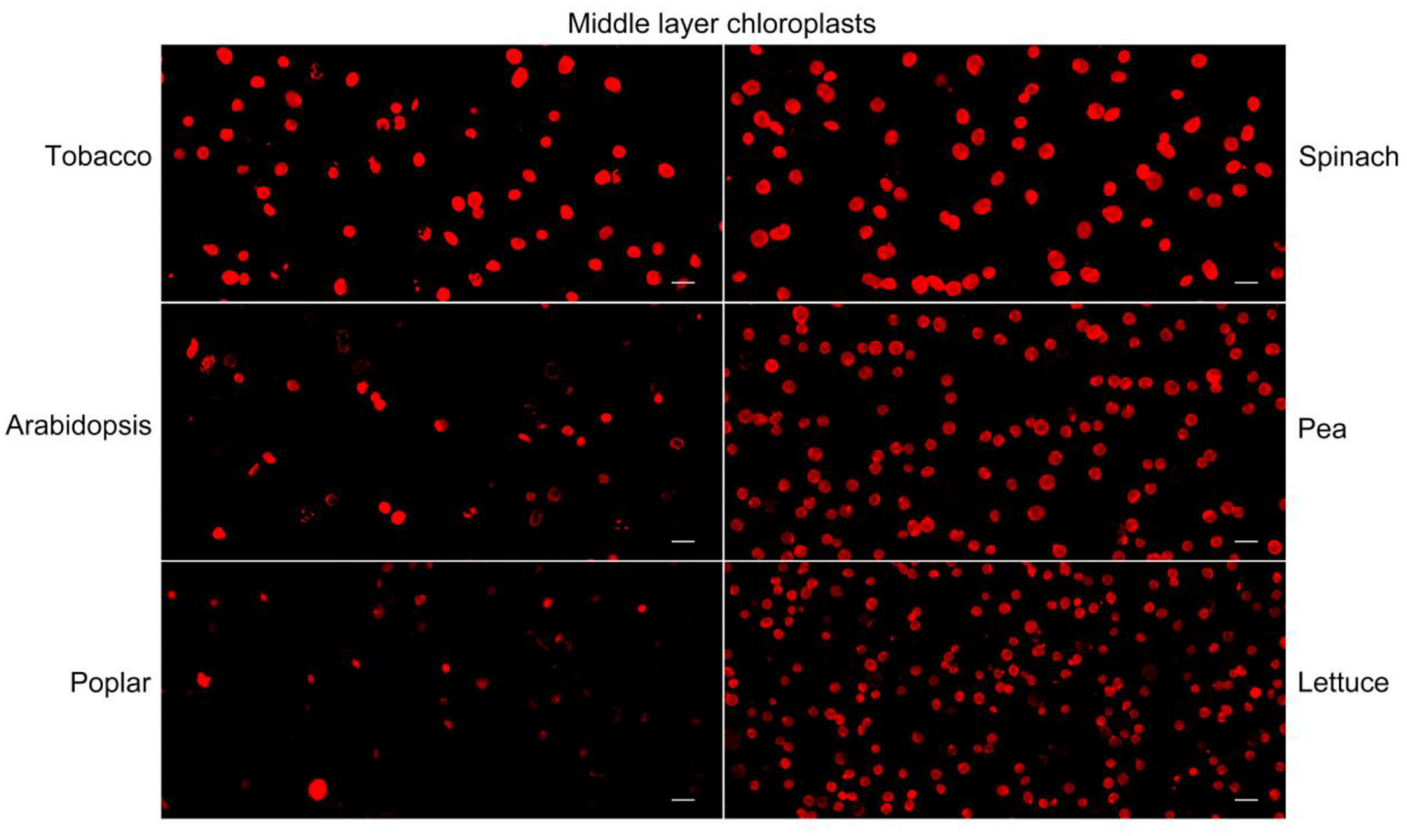
Nile red staining of middle layer chloroplasts isolated from various plants. Intact and broken chloroplasts can be distinguished by the integrity of their envelopes. Bar = 10 μm.

Nile red is a commonly used lipophilic dye that shows strong red fluorescence at the excitation wavelength of 550 nm (Fowler and Greenspan, 1985; Greenspan et al., 1985; Rumin et al., 2015). We test our method with Nile red as the dye on chloroplasts isolated from tobacco and spinach first as before (Figures 1, 4). Most of the chloroplasts from the middle layer have a relatively uniform fluorescence with clear and smooth boundaries, suggesting they were intact. In contrast, most of the chloroplasts from the upper layer were stained as small bright spots, suggesting they were broken. Then the chloroplasts isolated from other plants, including Arabidopsis, pea, poplar, and lettuce, were also stained with Nile red to see the effect of envelope membrane staining (Figure 5). The results were similar to that of Rhodamine staining (Figures 2, 3). As we can see, most of the chloroplasts from the middle layer of tobacco, spinach, pea and lettuce were intact, and a small part of the chloroplasts were broken. For the chloroplasts from the middle layer of Arabidopsis and poplar, most of them were broken. Overall, the staining results of Nile red looks to be similar to those of Rhodamine. Thus, Nile red can also be used as a dye like Rhodamine to stain the envelope membrane of chloroplasts to check their intactness.

### Chloroplasts From the Middle Layer May Be Mostly Broken

**Figure 6:**
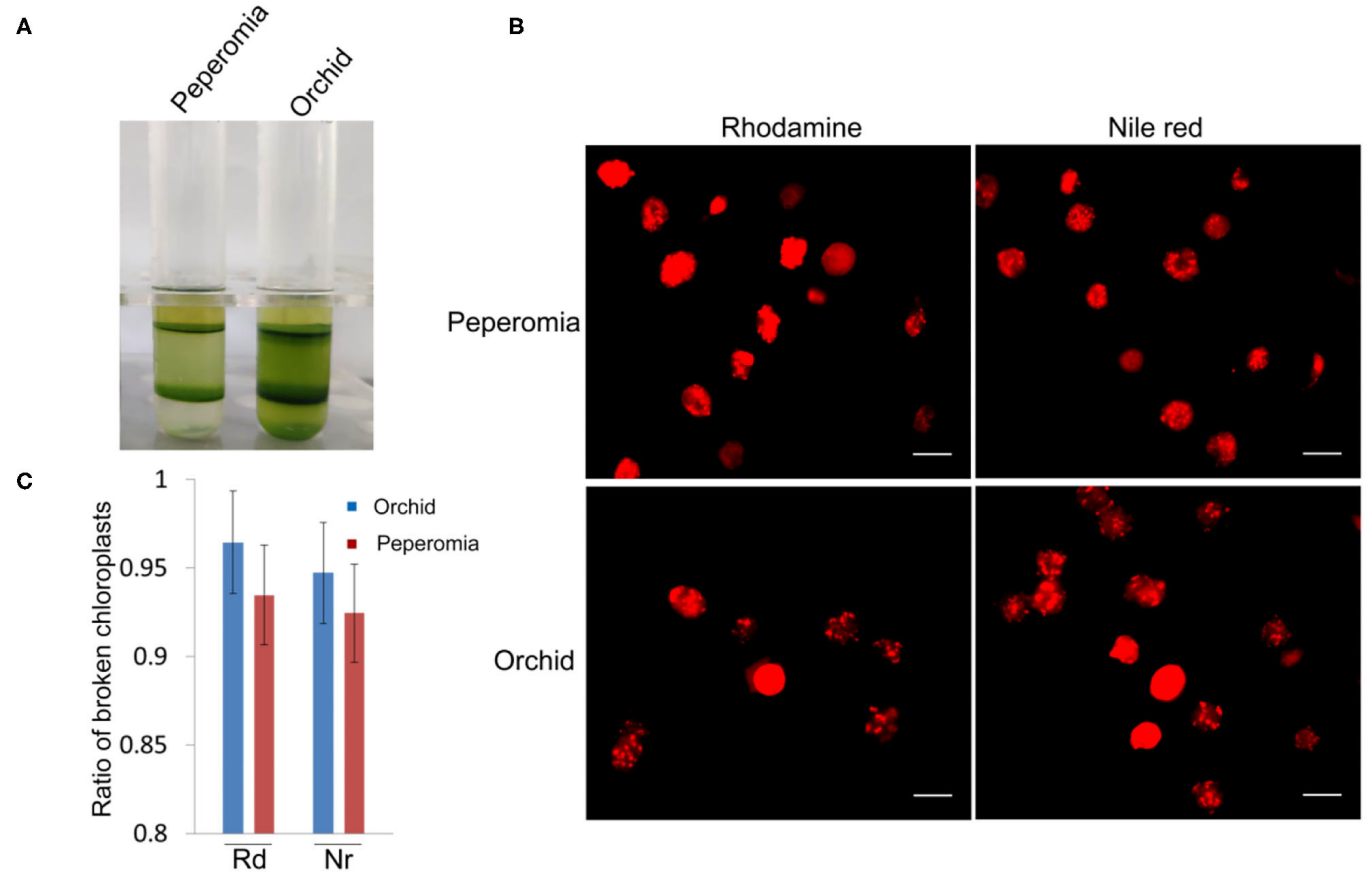
Analysis of the intactness of middle layer chloroplasts from peperomia and orchid. **(A)** Chloroplasts were separated into upper layer and middle layer by Percoll density gradient centrifugation. **(B)** Chloroplasts from the middle layer were stained with Rhodimine and Nile red, and observed with a fluorescence microscope. Bar = 10 μm. **(C)** Statistical analysis of the intactness of middle layer chloroplasts isolated from peperomia and orchid. Chloroplasts stained by Rhodamine or Nile red were photographed and analyzed. Rd, Rhodamine; Nr, Nile red. Error bars represent standard deviation.

As shown above, isolated chloroplasts from the middle layer of Arabidopsis and poplar were mostly broken (Figures 3, 5). We also isolated chloroplasts from other plants, such as peperomia and orchid. The isolated chloroplasts were separated well into upper layer and middle layer (Figure 6A). However, after being stained with Rhodamine and Nile red, most of those chloroplasts were found to be broken (Figure 6B). This was also confirmed by the statistical analysis, which showed that more than 90% of those chloroplasts were broken (Figure 6C). These data further suggest that checking the integrity of chloroplast envelope is necessary and our method is good at this.

## Discussion

Chloroplasts are involved in a variety of important biological processes in plants. Isolated chloroplasts are widely used in the study of chloroplasts, such as biochemical, physiological and cellular experiments (Gao et al., 2003, 2006; Baginsky and Gruissem, 2004; Lopez-Juez, 2007). It is essential to obtain intact chloroplasts in these experiments. Direct microscopy observation with bright field, phase contrast and autofluorescence might be used to check their intactness. However, we found this often could be misleading and cause serious problem to the experiments. For the chloroplasts isolated from plants in extended darkness, thylakoids will shrink and the envelope may look clear, and their intactness probably can be judged this way. However, in most cases, chloroplasts were isolated from healthy plants or plants grown under normal growth condition, they were almost fully filled with thylakoids and looked to have good shapes under microscope even if their envelopes were broken (Figure 3B; Supplementary Figure 1).

Although the intactness of chloroplasts can be checked by biochemical methods such as Coomassie blue staining or immuno-blot analysis of chloroplast proteins (Figures 2, 3) (Seigneurin-Berny et al., 2008a), it is relatively time consuming to obtain the results. Hill reaction method is a fast and simple method to estimate the intactness of chloroplasts based on biochemical reaction (Mills and Joy, 1980). In this study, we developed a method that use two dyes, Rhodamine or Nile red, with strong red fluorescence to stain the chloroplast envelope membrane and then show its integrity in several minutes. It can easily distinguish intact and broken chloroplasts by direct microscopy observation. It can also obtain a ratio of the intact or broken chloroplasts in a sample of isolated chloroplasts through statistical analysis (Figures 2, 3). The results of this method were validated by traditional biochemical methods (Figures 2, 3). In contrast to the traditional biochemical methods, it is simple, fast and accurate. Therefore, our method is very useful. In this study, we have tested our method only in several plants, but it could be used in many other plants.

It is usually believed that the chloroplasts in the middle layer of Percoll density gradient are intact (Seigneurin-Berny et al., 2008a,b), since chloroplasts in the upper layer are mostly broken. In many cases, this could be true. In our experiments, with our chloroplast staining method, we found that chloroplasts in the middle layer of Percoll density gradient could also be mostly broken (Figures 3, 6). Percoll density gradient centrifugation method mainly relies on the density of chloroplasts, so broken chloroplasts with higher density could stay in the middle layer. But their integrity can be easily revealed by our method to avoid mistakes in the experiment. Since chloroplasts from different plants could be in different physiological conditions, their isolation methods may not always be same. Hence, our method can also be used to quickly optimize the protocol for chloroplast isolation.

## Data Availability Statement

The original contributions presented in the study are included in the article/Supplementary Material, further inquiries can be directed to the corresponding author.

## Author Contributions

HG designed the experiments. JA, XM, LW, and XuL carried out the experiments. XiL, JA, and HG prepared the manuscript. All authors contributed to the article and approved the submitted version.

## Conflict of Interest

The authors declare that the research was conducted in the absence of any commercial or financial relationships that could be construed as a potential conflict of interest.
