# Supplementary material for: Visualizing the Integrity of Chloroplast Envelope by Rhodamine and Nile Red Staining
Source: Front Plant Sci. 2021 Apr 26;12:668414. doi: 10.3389/fpls.2021.668414 (PMC8107281; doi:10.3389/fpls.2021.668414)
Supplement: Supplementary file 1 [file Image_1.pdf]

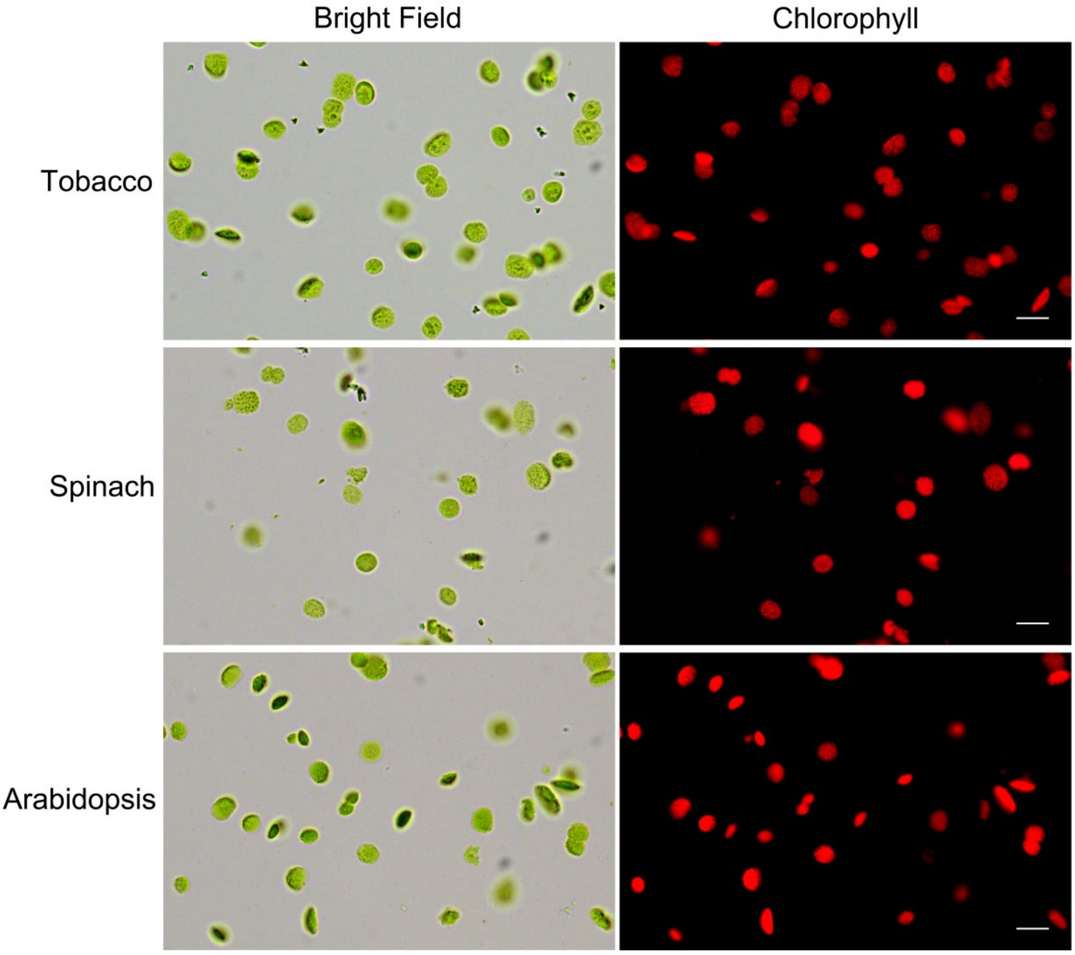

Supplementary Figure 1 | Isolated chloroplasts observed with a bight field and fluorescence microscope. Chloroplasts were isolated from tobacco, spinach and Arabidopsis leaves through Percoll density gradient centrifugation. Bar = 10 µm.
